# Taking Charge: Metal Ions Accelerate Amyloid Aggregation in Sequence Variants of α-Synuclein

**DOI:** 10.1021/jasms.2c00379

**Published:** 2023-02-16

**Authors:** Emily J. Byrd, Martin Wilkinson, Sheena E. Radford, Frank Sobott

**Affiliations:** † Astbury Centre for Structural Molecular Biology, School of Molecular and Cellular Biology, Faculty of Biological Sciences, University of Leeds, Leeds LS2 9JT, United Kingdom

## Abstract

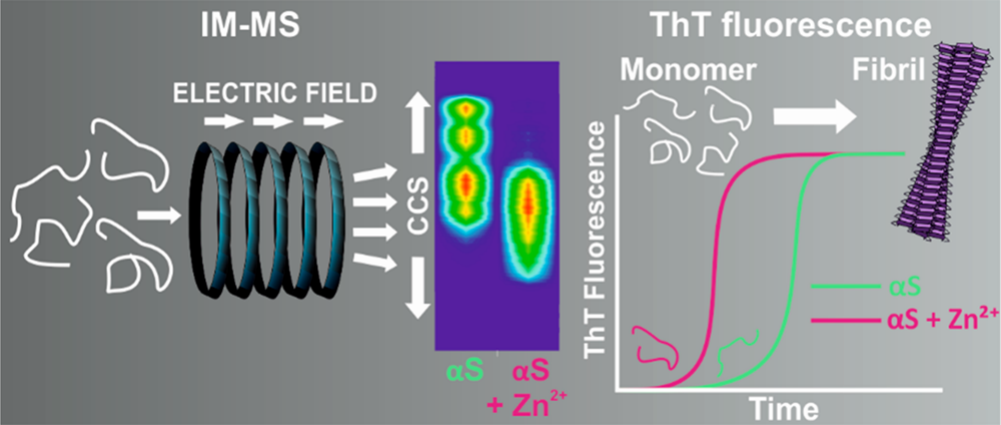

Αlpha-synuclein (αS) is an intrinsically disordered protein which exhibits a high degree of conformational heterogeneity. *In vivo*, αS experiences various environments which cause adaptation of its structural ensemble. Divalent metal ions are prominent in synaptic terminals where αS is located and are thought to bind to the αS C-terminal region. Herein, we used native nanoelectrospray ionization ion mobility-mass spectrometry to investigate changes in the charge state distribution and collision cross sections of wild-type N-terminally acetylated (NTA) αS, along with a deletion variant (ΔΔNTA) which inhibits amyloid formation and a C-terminal truncated variant (119NTA) which increases the rate of amyloid formation. We also examine the effect of the addition of divalent metal ions, Ca^2+^, Mn^2+^, and Zn^2+^, and correlate the conformational properties of the αS monomer with the ability to aggregate into amyloid, measured using Thioflavin T fluorescence and negative stain transmission electron microscopy. We find a correlation between the population of species with a low collision cross section and accelerated amyloid assembly kinetics, with the presence of metal ions resulting in protein compaction and causing ΔΔ to regain its ability to form an amyloid. The results portray how the αS conformational ensemble is governed by specific intramolecular interactions that influence its amyloidogenic behavior.

## Introduction

The amyloid precursor protein alpha-synuclein (αS) is structurally defined as an intrinsically disordered protein (IDP) and visits a large and diverse conformational space, including partially compact and extended states.^1^ Aggregation and self-assembly of αS into β-sheet rich amyloid fibril structures is associated with the onset of Parkinson’s disease (PD) and other synucleopathies.^2,3^ The neuropathology of PD is characterized by the deposition of insoluble cellular inclusions called Lewy Bodies (LB) in dopaminergic neurons in the substantia nigra of the brain.^4,5^ The composition of LBs consists of fibrillar αS, lipids, mitochondria, metal ions, and various other cellular components.^6−8^ Monomeric αS is found *in vivo* to partition between the cytoplasm and phospholipid membranes.^9−11^ Although the exact functional role(s) of αS remain uncertain, localization of αS to presynaptic nerve terminals suggests a role in vesicle binding, clustering, and neurotransmitter release.^12−15^ This proposed function relies on the lipid binding properties of αS which are governed by the charge distribution across the protein sequence.^16−18^

The sequence of αS is divided into three distinct regions: the N-terminal region which overall is positively charged (residues 1–60) containing imperfect KTKEGV repeats responsible for lipid binding,^16^ the nonamyloid-β component (NAC) core (residues 61–95) which is hydrophobic and amyloidogenic, forming the core of αS fibril polymorphs,^19−23^ and the C-terminal region (residues 96–140) which contains 14 negatively charged residues at physiological pH responsible for binding dopamine, calcium, and other metal ions.^24−26^

The conformational freedom exhibited by αS may play a role in its aggregation pathway, with different monomeric conformation families possessing different amyloid-forming potential.^27^ Recently, Brodie et al. used cross-linking mass spectrometry (MS) and single molecule Förster resonance energy transfer (smFRET) guided molecular dynamics simulations to portray possible conformations of monomeric αS.^28^ Their findings identified the importance of inter-residue contacts between the N- and C-terminal regions for stabilizing conformations, proposing that a transient β-hairpin structure involving the NAC and C-terminal regions may form a nucleation site for early oligomers which could initiate the formation of the cross-β structure of fibrils. Further to this, Chen et al. used these same smFRET data as an experimental restraint to guide discrete molecular dynamics (DMD) simulations in order to generate a snapshot of the αS conformational ensemble.^29^ They showed that the ensemble of monomeric αS structures can be split into eight clusters distinguished by conformation, some of which exist within nanosecond and microsecond time scales. Transitions between conformations may follow a hierarchy of subpopulation dynamics, whereby some subpopulations may exist long enough to facilitate ligand binding. Specific subpopulations of monomeric αS structures may preferably facilitate amyloid assembly and ultimately PD progression.

Oligomers that are believed to be toxic^30^ and amyloid fibril structures of αS are both composed primarily of β-sheets.^31,32^ The adoption of specific structures under defined conditions infers that the roles of αS, be they functional or pathological, might be a result of the precise conformational species of αS monomers which can transition into β-sheet structures as oligomers assemble. Therefore, it is crucial to determine how the conformational ensemble of monomeric αS and its sequence variants relates to their amyloid-forming potentials. Characterizing transient and heterogeneous species of IDPs, however, is experimentally challenging. Structural techniques such as nuclear magnetic resonance (NMR) rely on population averaging to visualize conformations in dynamic equilibrium with one another, which limits this technique in the number of states that it can discriminate.^33^ Hence, the data output struggles to capture the structural fingerprints of species in a broad ensemble of interconverting states. Here, an integrative approach using native nanoelectrospray ionization (nESI) ion mobility-mass spectrometry (native IM-MS), measurement of the kinetics of amyloid formation using thioflavin T (ThT) fluorescence, and negative stain transmission electron microscopy (TEM) has been applied in order to capture the αS conformational ensemble under different conditions and relate this fingerprint to its amyloid assembly kinetics.

Variants of αS with deletions in the N-terminal region were recently created in order to identify regions within the 140 amino acid protein chain that could be important in controlling its amyloid formation propensity.^34,35^ Doherty et al. used *in silico* analysis to identify two regions in the N-terminal region of αS that exhibit predicted low solubility and high aggregation propensity. These regions include the seven residue sequence ^36^GVLYVGS^42^ named P1 and the 13 residue sequence ^45^KEGVVHGVATVAE^57^ named P2. Deleting P1 to create the αS variant ΔP1 inhibited aggregation into amyloid at physiological pH (pH 7.5) but not at acidic, lysosomal pH (pH 4.5). Deleting P2 alone (ΔP2) had little effect on the half time (*t*_50_) of amyloid formation. However, deleting both P1 and P2 in tandem (ΔΔ) abolished amyloid formation *in vitro* at both physiological and lysosomal pH within 100 h as shown by ThT fluorescence.^34,35^ How these deletions control or abolish amyloid formation, however, remained unclear. Deleting these seemingly critical N-terminal sequences may disrupt intramolecular interactions within the protein chain which may affect the ability of αS to achieve an amyloid competent conformation in which the NAC region is sufficiently exposed. Indeed, cryogenic electron microscopy (cryoEM) structures of αS fibril polymorphs have identified residues in the P2 region as important stabilizers of its fibril cores. For example, residues ^47^GVVHGVATVA^56^ containing uncharged glycine and alanine residues are shown to form the steric zipper between protofilaments.^21^ Residues ^50^HGVATVAE^57^ were also revealed by cryoEM to form a dimer interface between αS subunits in the fibril structure, forming a parallel steric-zipper configuration,^22,36^ while the P2 region is part of the core in all of the 48 currently solved amyloid structures of αS.^37^ Native IM-MS offers the potential to better understand how the conformational heterogeneity of ΔΔ αS correlates with its amyloid assembly propensity and whether removing the P1 and P2 regions which contain in total one positive lysine residue, the singular histidine residue present in αS, and two negatively charged residues disrupts the overall charge balance of the αS sequence.

**Figure 1:**
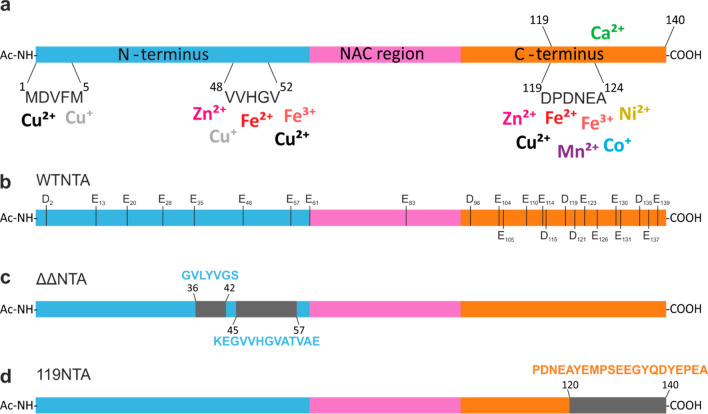
αS is predicted to bind metal ions primarily in the negatively charged C-terminal region. (a) A range of monovalent, divalent, and trivalent metal ions are known to bind to negatively charged aspartic and glutamic acid residues that are enriched in the C-terminal region of αS, with the binding sites mapped by NMR.^24^ Some transition metals also interact with His50 and methionine residues in the N-terminal region.^48^ (b) Full length sequence of WT NTA αS acetylated at the N-terminus (denoted by Ac-NH) with each glutamic acid and aspartic acid residue labeled across the entire sequence. (c) Sequence of the ΔΔ*N*-terminally acetylated αS variant, the P1 and P2 regions, which are removed in this construct, are indicated by black boxes corresponding to residues 36–42 and 45–57, respectively. (d) Sequence of the C-terminal truncation variant cleaved at residue 119 in which residues 120–140 are deleted, as indicated by a black box. Variant 119 was also N-terminally acetylated in this study.

*In vivo*, αS is predominantly acetylated at the N-terminus.^38^ More specifically, the composition of Lewy Body deposits has been shown to contain a high degree of N-terminally acetylated (NTA) αS.^39,40^ NTA of αS has been shown to influence its membrane binding affinity through increased helicity of the N-terminal region, as well as to facilitate chaperone binding.^11,41^ αS *in vivo* is located in presynaptic nerve terminals in the brain. Calcium ions (Ca^2+^) are known to bind extensively to αS at the C-terminal region;^42,43^ they are essential for the transduction of electrical signals into chemical signals, but other metal ions have been shown to alter synaptic transmission.^44,45^ Further to this, heavy metal ions have been linked to neurotoxicity and are markedly elevated in amyloid plaques deposited in the brain.^45−47^ In its native environment, αS will thus experience different physiological environments and conditions which could potentially alter the distribution of monomeric conformers and their propensity to aggregate into amyloid. Due to the high abundance of negatively charged residues in the C-terminal region of αS, binding of positively charged ions is observed in this region (Figure 1a).^24,25^ Binding of metal ions to residues in the C-terminal region will affect the charge distribution of αS through neutralization of Glu and Asp residues, although precisely how interactions with different metal ions alters the monomeric conformational ensemble of αS is unclear.

Here, the binding of divalent ions Ca^2+^, Mn^2+^, and Zn^2+^ to N-terminally acetylated wild-type (WTNTA), ΔΔNTA, and a C-terminally truncated variant, 119NTA αS (Figure 1), has been observed through native MS, and the resultant effects on the conformational ensemble and amyloid assembly propensity have been investigated using native IM-MS, ThT fluorescence, and TEM, in order to correlate the conformational dynamics of αS with amyloid formation. The results provide evidence which suggests that perturbing the charge distribution of αS through metal ion binding, or sequence truncations, restricts the conformational freedom of the polypeptide chain and causes population shifts in the conformational ensemble, which has marked effects on the rates of amyloid assembly. Such compaction of monomeric αS species may uncover the earliest stages of amyloid assembly at the monomer level, potentially identifying whether the enhancement of particular conformations predispose the amyloid propensity of αS.

## Materials and Methods

### Protein Expression and Purification

Competent BL21 DE3 cells expressing NatB acetylase were prepared as follows. BL21 DE3 (Agilent) cells were transformed with the pNatB plasmid (Addgene 53613), and a single colony was used to inoculate a starter culture of LB media overnight at 37 °C, 200 rpm. The overnight culture was used to inoculate 500 mL LB containing 25 μg/mL chloramphenicol until an OD_600_ of 0.6 was reached. Cells were pelleted at 4500*g* for 5 min. Cells were resuspended in 30 mM potassium acetate, 10 mM CaCl_2_, 50 mM MnCl_2_, 100 mM RbCl, 15% (v/v) glycerol, pH 5.8. Cells were incubated on ice for 5 min before pelleting and further resuspension in 10 mM MOPS, 75 mM CaCl_2_, 10 mM RbCl, 15% (v/v) glycerol, pH 6.5. Competent cells were stored at −80 °C until used.

Competent NatB-BL21 DE3 cells were transformed with a pET23a plasmid encoding WT human full length αS, ΔΔ or 119 αS to express both NatB and αS for N-terminal acetylation. Expressed protein was purified by cell lysis in 25 mM Tris-HCl pH 8.0, 100 μg/mL lysozyme, 50 μg/mL PMSF, 1 mM benzamidine, and 20 μg/mL DNase and homogenized using an IKA T 18 ULTRA-TURRAX homogenizer (IKA, Staufen, Germany). The lysate was heated to 80 °C for 10 min and then centrifuged at 35,000*g* for 30 min, 4 °C, followed by ammonium sulfate precipitation (50% w/v). The pellet containing αS was diluted in 20 mM Tris-HCl, pH 8.0, and purified by anion exchange using a 350 mL Q-Sepharose fast flow anion-exchange column on an ÄKTA Prime (Cytiva, UK). Bound αS was eluted in a gradient of 0–500 mM NaCl, in 20 mM Tris-HCl, pH 8.0, over a volume of 700 mL. Fractions containing αS were dialyzed against 5 × 5 L of 50 mM ammonium bicarbonate (3500 MWCO) at 4 °C and lyophilized. Freeze-dried protein was resuspended in 50 mM ammonium bicarbonate at 5 mg/mL and loaded onto a HiLoad 26/60 Superdex-75 column for size-exclusion chromatography. Fractions containing αS were pooled and lyophilized.

### Kinetics of Amyloid Formation

Kinetics of αS amyloid formation were monitored in a 96-well, nonbinding, flat-bottom, half-area microplate (Corning, USA; 10438082) containing one Teflon polyball (1/8″ diameter; Polysciences Europe, Eppelheim, Germany) per each well of sample. Samples of 100 μL containing 100 μM αS with 20 μM Thioflavin T in 20 mM ammonium acetate, pH 7.5, were incubated at 37 °C shaking at 600 rpm in a FLUOstar omega plate reader (BMG Labtech, Ortenburg, Germany). Fluorescence intensity was measured by exciting at 440 ± 10 nm and collecting emission at 482 ± 12 nm using a bandpass filter. For experiments involving the addition of metal ions, zinc acetate, manganese acetate, or calcium acetate (Sigma Life Sciences, Germany) was added at a concentration of 2.5 mM (i.e., 25 fold excess over protein) per well. Results were blank-corrected using wells containing 20 μM ThT in 20 mM ammonium acetate, pH 7.5, and normalized to the maximum fluorescence value of each curve.

### Negative Stain TEM

A sample of 5 μL was taken from the ThT plate at the end-point of each reaction, loaded onto a glow discharged (30s, Pelco Easi-glow), 400 mesh continuous carbon grid, and incubated for 2 min. The sample was blotted and washed twice with H_2_O before being blotted and stained twice with 2% (w/v) uranyl acetate. Grids were imaged on a Tecnai F20 electron microscope (FEI) with a Ceta CCD detector (FEI) in the Astbury EM facility (University of Leeds), using a nominal magnification of 9600× corresponding to a pixel size of 1.05 nm/pixel.

### Quantification of Fibril Yield

Fibril yields were determined by pelleting 50 μL of the end point of the ThT reaction at 100,000*g* (Optima TLX ultracentrifuge, Beckman Coulter, TLA 100 rotor) at 4 °C for 30 min, and the amount of protein in the sample, as well as an unclarified sample from the reaction end point, was quantified by densitometry of sodium dodecyl sulfate–polyacrylamide gel electrophoresis (SDS–PAGE) gels. Tris-tricine buffered 30% (w/v) acrylamide:0.8% (w/v) bis(acrylamide) gels were stained with InstantBlue Coomassie/protein stain and imaged on an Alliance Q9 imager (Uvitec, Cambridge, UK). Band intensities were quantified using ImageJ 1.52a.

### Native IM-MS

Native IM-MS experiments were performed on a Synapt G1 HD mass spectrometer (Waters Corporation, Wilmslow, UK) with traveling (T-wave) ion mobility and a nano-ESI source using in-house generated gold- and palladium-coated capillaries. αS variants were analyzed at a concentration of 20 μM, and spectra were collected with and without the addition of 500 μM zinc acetate, manganese acetate, or calcium acetate (Sigma Life Sciences, Germany) at a ratio of 1:25 αS:metal ion. The unbound peak profiles for all three variants were taken from an external control without metal, since the unbound peak was sometimes not visible in spectra with a 25-fold excess of metal ion. MassLynx V4.1 (Waters Corporation, Wilmslow, UK) was used for data processing. Instrument parameters were set at capillary voltage 1.4 kV, source temperature 30 °C, sampling cone 18 V, extraction cone 1.0 V, trap collision energy 5.0 V, transfer collision energy 2.0 V, trap DC bias 30 V, IM wave velocity 300 m/s, and IM wave height 7.0 V. Gas pressures in the instrument were trap cell 0.0256 mbar and IM cell 0.36 mbar. The IM data was calibrated according to the Bush database^49^ using denatured cytochrome c (charge states 13+ to 19+), myoglobin (charge states 15+ to 24+), and ubiquitin (charge states 7+ to 13+) at 10 μM in 50% (v/v) acetonitrile, 0.1% (v/v) formic acid.

## Results

### Divalent Metal Ions Increase the Rate of Amyloid Assembly

**Figure 2:**
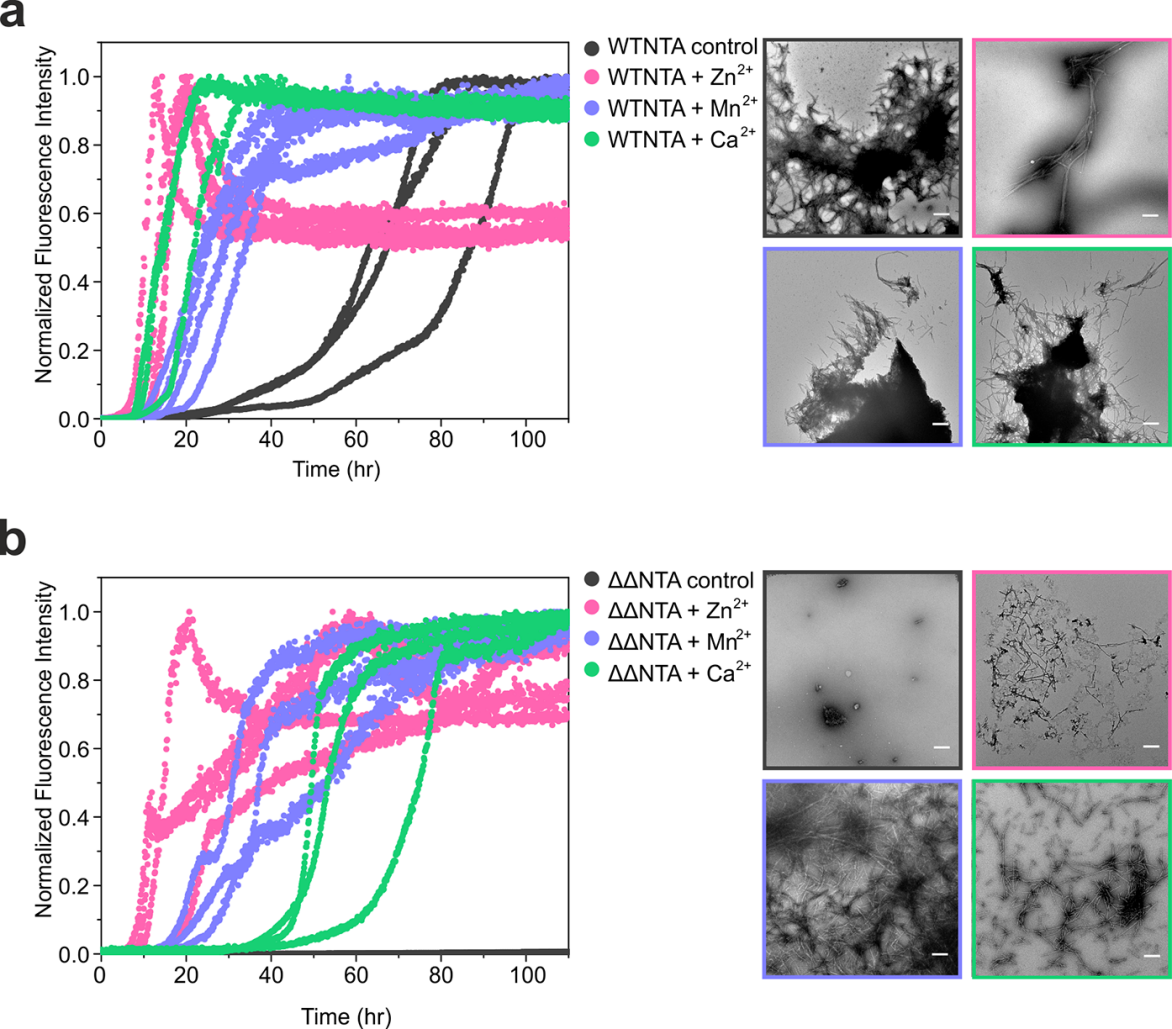
Metal ions increase the rate of WTNTA αS amyloid asssembly and switch on amyloid formation of ΔΔNTA αS. (a) Amyloid assembly of three replicates of WTNTA αS (gray) alone and in the presence of a 25-fold molar excess of Zn^2+^ (pink), Mn^2+^ (purple), or Ca^2+^ (green). Negative stain TEM images are shown as boxes (right) colored according to the respective ThT curves. All of the images were taken at the end of the ThT reactions using the same magnification. The scale bar corresponds to 300 nm in all images. (b) As in panel (a), but for ΔΔNTA αS.

To investigate how the addition of the metal ions Zn^2+^, Mn^2+^, and Ca^2+^ affects the aggregation propensity of WTNTA αS and ΔΔNTA αS, the rate of amyloid assembly was measured using ThT fluorescence (Figure 2a,b). The end points of the reaction (after 110 h) were imaged via negative stain TEM, and the percentage of pelletable material was determined through ultracentrifugation and quantitative analysis of the percent pelleted material by analysis using SDS–PAGE.

For these experiments, a 25-fold molar excess of metal ion:αS was used in order to saturate possible binding sites, following previous protocols.^50^ The rate of ThT positive amyloid assembly for WTNTA αS was accelerated when each of the three tested metal ions were added (Figure 2a). A significant decrease in the half time was observed, determined from *t*_50_ values which represent the time taken for each fluorescence signal to reach half of the plateau value calculated using AmyloFit 2.0.^51^ This effect is most prominent in the presence of Zn^2+^, where the *t*_50_ is reduced from an average of 64.4 ± 18.8 h to 13.4 ± 2.6 h (Figure S1). All of the tested ions are found in presynaptic nerve terminals;^45−47,52,53^ therefore, the amyloid assembly kinetics presented here could offer understandings of the behavior of αS in its native environment. In the absence of metal ions, the variant ΔΔNTA αS does not assemble into ThT positive amyloid fibrils within 110 h (Figure 2b, Figure S1). The deletion of both the P1 and P2 regions might disrupt intramolecular interactions which are involved in the assembly of a partially compact structure on pathway for amyloid assembly. Strikingly, the addition of Zn^2+^, Mn^2+^, or Ca^2+^ all induced amyloid formation of ΔΔNTA αS, which otherwise does not assemble into amyloid on the time scale of these experiments (Figure 2b).

The results presented in Figure 2b suggest that metal ion binding could enable the formation of new amyloid-competent conformation(s) adopted by the monomer of ΔΔNTA αS or result in the enhancement in the population of a pre-existing amyloid-competent subpopulation. Similarly to the observations with WTNTA αS, Zn^2+^ exerts the greatest effect on the rate of ThT positive amyloid assembly for ΔΔNTA αS, with Mn^2+^ less effective and Ca^2+^ having the smallest effect of the three metal ions tested. The resultant effect of each ion could arise through different binding stoichiometries or coordination sites resulting in different conformational shifts upon binding. The increase in the rate of amyloid formation could be a result of neutralization of the negatively charged C-terminal region of αS due to coordination of divalent metal ions, which disrupts the overall charge distribution across the protein sequence. This charge distribution could be a fine-tuned feature of αS, and disruption would result in exposure of the NAC region. The conformational properties of αS, therefore, were next explored by native IM-MS to gain insight into how αS conformational flexibility could influence amyloid propensity and the rate of assembly.

### Native IM-MS Compaction of Low Charge States Occurs When Metal Ions Bind αS

**Figure 3:**
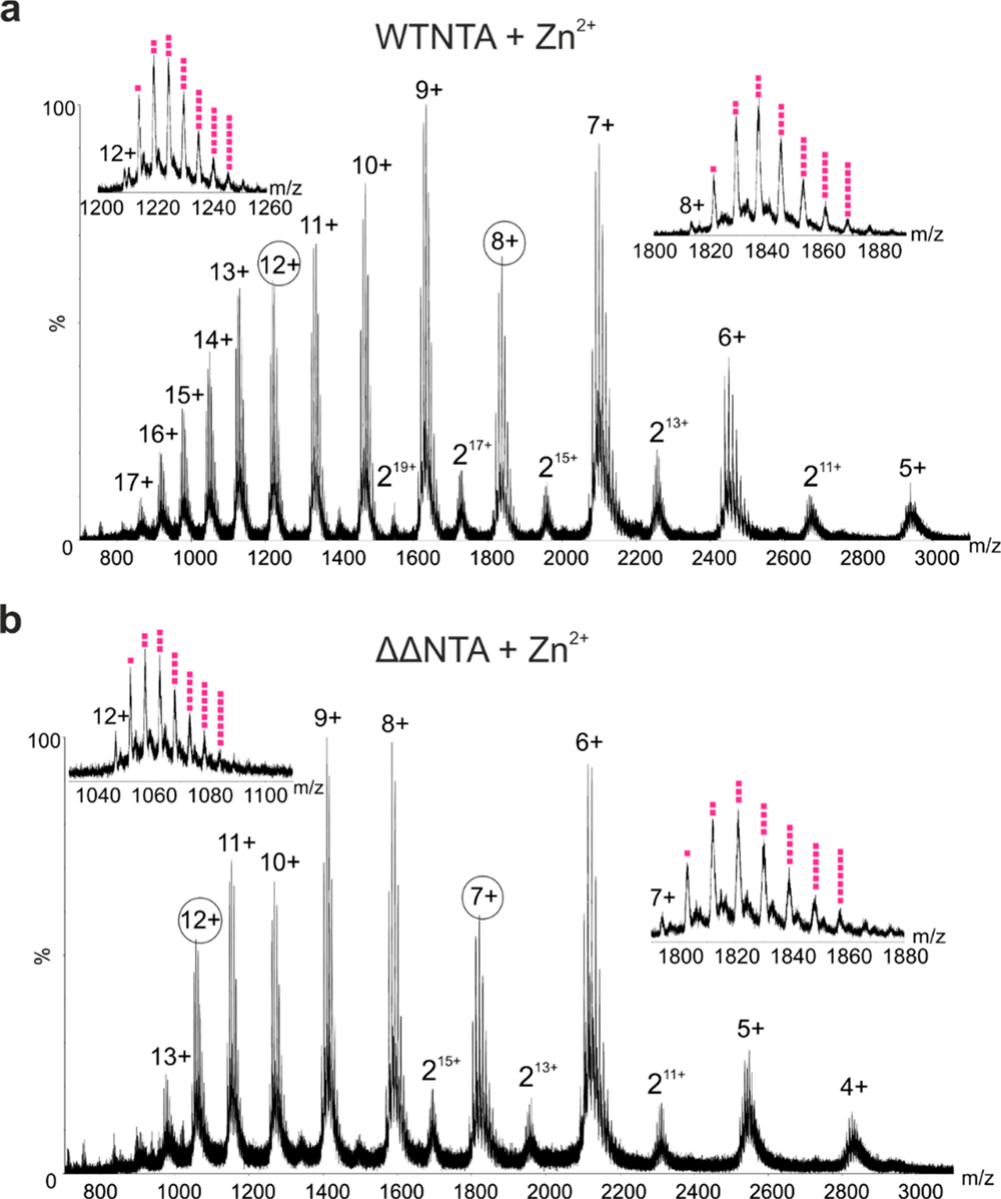
Native nESI mass spectra showing Zn^2+^ binding to WTNTA and ΔΔNTA αS. (a) Native nESI mass spectrum of WTNTA αS bound to Zn^2+^ ions. Insets show up to seven Zn^2+^ bound to the 8+ and 12+ charge states (pink squares). (b) As in panel (a) but for ΔΔNTA αS bound to Zn^2+^. Insets show up to seven metals bound to the 7+ and 12+ charge states. The protein concentration was 20 μM in 20 mM ammonium acetate, pH 7.5, at a molar ratio of 1:25 αS:Zn^2+^. Dimers are indicated by “2”. Spectra were acquired using a Synapt G1 instrument.

Ion mobility MS can detect subtle changes in compaction or expansion of dynamic protein conformations which may not already be visible from native charge state distributions (CSD). The CSD of WTNTA αS in the presence of Zn^2+^ (Figure 3; the protein in the absence of metal ion is shown in Figure S2) shows a multimodal distribution, with surprisingly low charge states (5+ to 9+) present for an IDP indicating the presence of compact conformations.^54,55^

**Figure 4:**
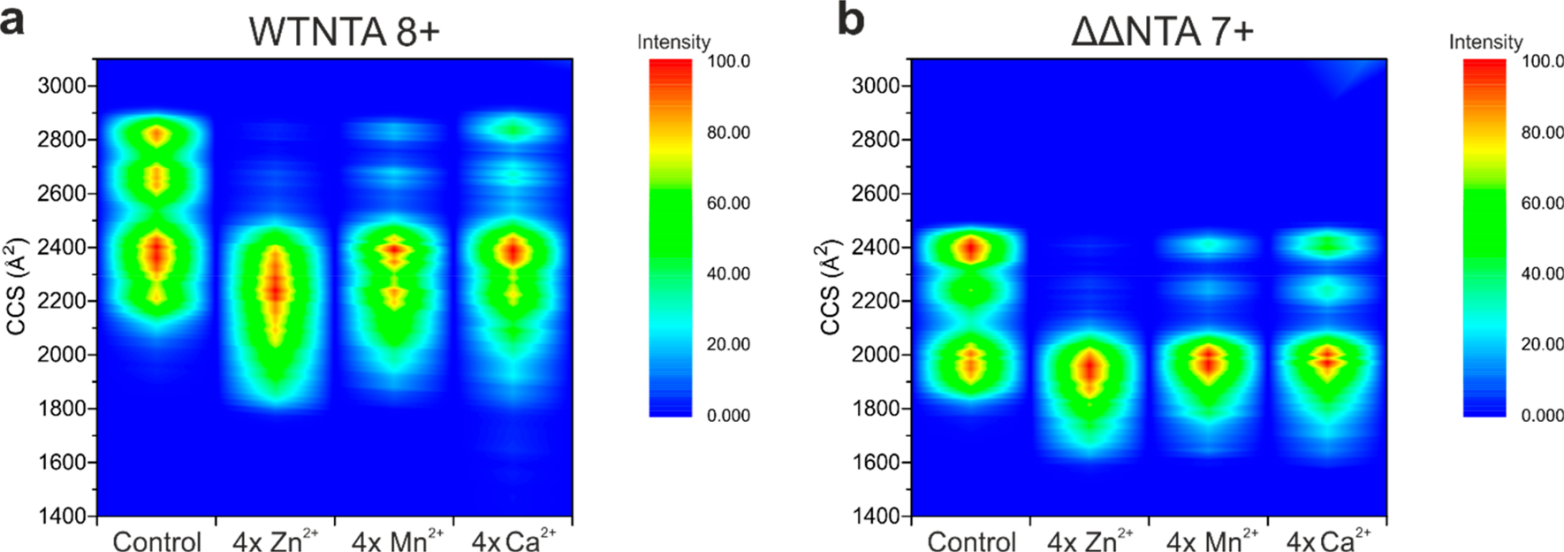
Native nESI-IM mass spectra showing compaction of WTNTA and ΔΔNTA αS when metal ions bind. (a) CCS fingerprints of the 8+ charge state of WTNTA αS either alone calculated using an external control or bound to four ions of either Zn^2+^, Mn^2+^, or Ca^2+^. (b) CCS fingerprints of the ΔΔNTA αS 7+ charge state either alone calculated using an external control or bound to four ions of either Zn^2+^, Mn^2+^, or Ca^2+^. All spectra were acquired using a protein concentration of 20 μM in 20 mM ammonium acetate, pH 7.5. A 25-fold molar excess of metal ion was added. CCS values were calculated using ATDs extracted from MassLynx 4.1 software and calibrated as described in the Methods section.

Binding of divalent metal ions to the intrinsically disordered ensemble of αS may result in significant structural changes due to metal ion coordination, most likely by aspartic acid and glutamic acid residues in the protein sequence. Analysis of WTNTA αS using native IM-MS results in a conformational fingerprint which contains four distinct conformations at the 8+ charge state, which was selected to represent the greatest conformational diversity of all charge states in the unbound state (Figure 4a), consistent with previous analysis of unacetylated WT αS.^50^ When metal ions Zn^2+^, Mn^2+^, or Ca^2+^ are added, a multimodal CSD is still observed (Figure 3, Figure S3) and a clear compaction of the polypeptide chain occurs (Figure 4a). Strikingly, in the case of ΔΔNTA αS, a similar compaction with divalent metal ions is seen (Figure 4b, Figure S4) for the 7+ charge state. We selected the 7+ charge state here as it has equivalent charge density on its proportionally smaller solvent-accessible surface compared with the 8+ charge state for the larger WTNTA αS. It also exhibited the greatest conformational diversity in the apo form, involving three distinct conformations for this deletion construct (Figure 4b). In particular, the CSD (Figure 3b) and compaction effect (based on the relative population of each species observed in the IM-MS CCS data) is strongest in the presence of Zn^2+^ (Figure S5), which also resulted in the fastest amyloid assembly kinetics.

**Figure 5:**
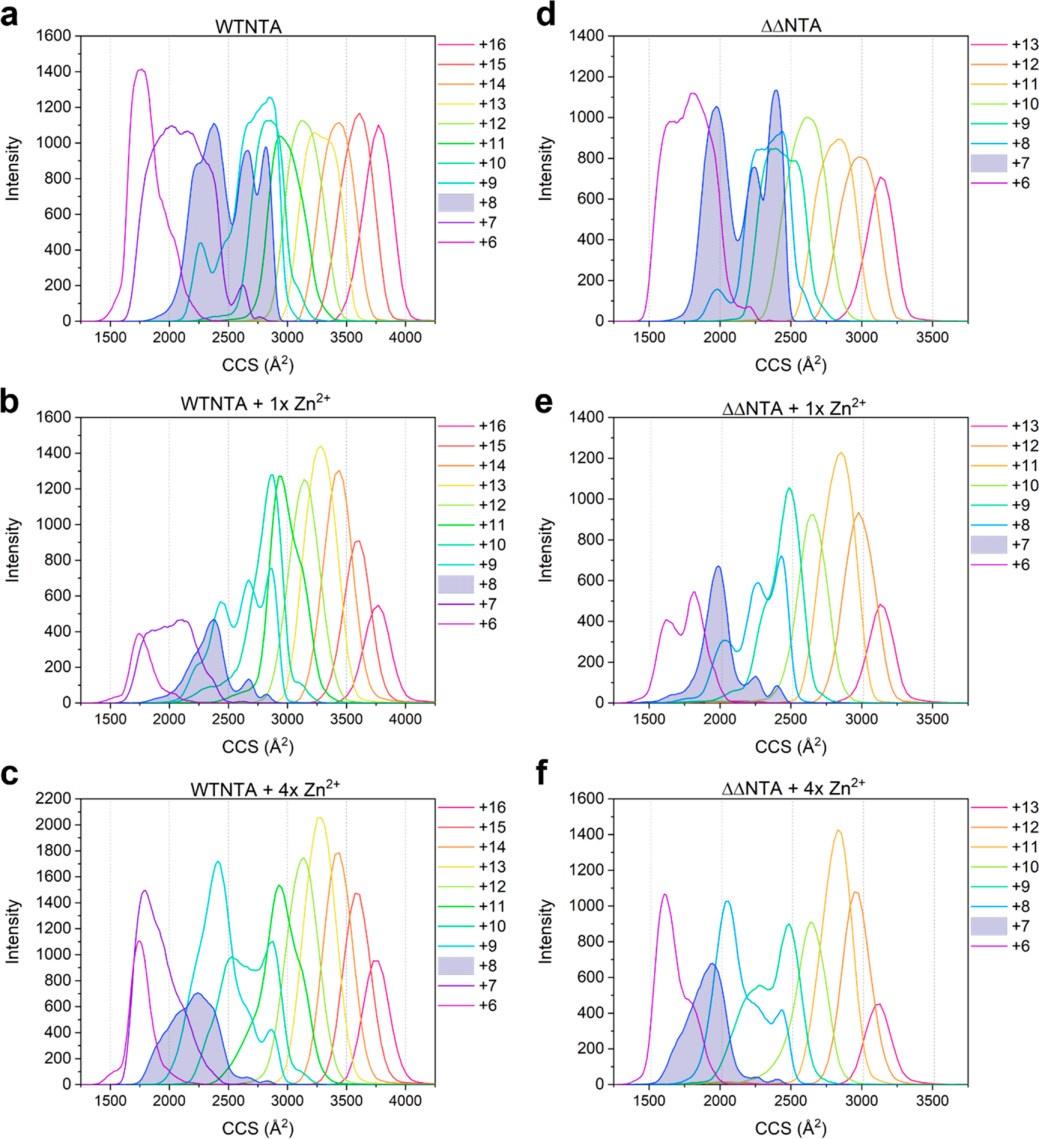
Metal ions selectively modulate compaction of low charge states of the αS variants. CCS fingerprints of the entire charge state distribution (6+ to 16+) of (a) WTNTA αS alone, (b) WTNTA αS bound to one Zn^2+^, and (c) WTNTA αS bound to four Zn^2+^ ions. The 8+ charge state is highlighted by shading in each plot. The spectra show that lower charge states of WTNTA αS become more compact when Zn^2+^ binds, with higher charge states being relatively unaffected by Zn^2+^ binding. CCS fingerprints of the entire charge state distribution (6+ to 13+) of (d) ΔΔNTA αS alone, (e) ΔΔNTA αS bound to one Zn^2+^, and (f) ΔΔNTA αS bound to four Zn^2+^ ions. The 7+ charge state is highlighted in d–f where a similar effect can be observed to WTNTA αS. ATDs were extracted using MassLynx 4.1.

When studying conformational changes by IM-MS, it is important to consider all charge states from the broad, multimodal distribution, as this reflects the entirety of the conformational ensemble.^54^ The native nESI mass spectra of WTNTA and ΔΔNTA αS show binding of Zn^2+^ to all charge states to a similar extent at the molar excess used (Figure 3a,b). Since Zn^2+^ exhibited the greatest accelerator effect on amyloid formation and greatest effect on the extent of compaction with metal ion addition for both variants, this ion was selected for analysis of CCS effects. The CCS fingerprint for each charge state is plotted in Figure 5a–c for WTNTA αS and Figure 5d–f for ΔΔNTA αS, each unbound, bound to one Zn^2+^, and bound to four Zn^2+^. Investigating the effect of Zn^2+^ binding on the CCS values reveals that structural remodelling of αS primarily occurs at low charge states. Low charge states (6+ to 9+ for WTNTA and 6+ to 8+ for ΔΔNTA αS) represent compact conformations with a smaller solvent accessible surface area (SASA), reducing the amount of protonation during native ESI. These compact conformations become even more compact upon Zn^2+^ binding, whereas ions with higher charge states reflecting a larger SASA (more extended conformations) exhibit no such striking change in CCS in response to binding of one or four Zn^2+^ ions. Zn^2+^ exhibits specific conformational effects on particular αS conformations, likely due to facilitated coordination of binding due to the close proximity of negatively charged residues in the C-terminal region, together with the presence of some negatively charged residues, and transition metal coordinating methionine and His50 residues in the N-terminal region (Figure 1b).

### C-Terminal Truncation Variant 119NTA of αS Retains Metal Ion Binding

**Figure 6:**
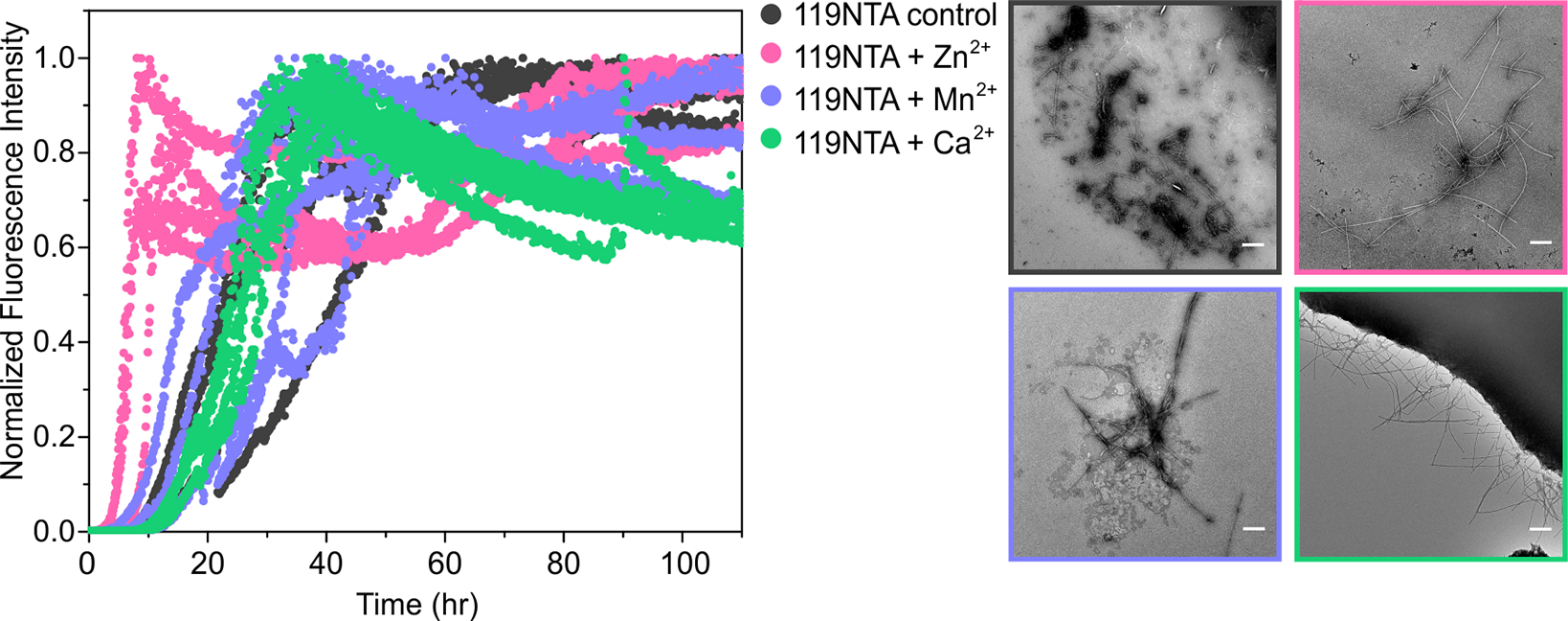
Metal ion binding modulates the rate of 119NTA αS amyloid asssembly. Amyloid assembly of 119NTA αS (black) alone or in the presence of Zn^2+^ (pink), Mn^2+^ (purple), or Ca^2+^ (green). Negative stain TEM images taken at the end of the reactions are shown (right) with box outlines colored according to the respective ThT curves. The scale bar corresponds to 300 nm for all images.

Within Lewy bodies, around 15% of total αS is estimated to be truncated within the C-terminal region, resulting in different length variants of αS which are naturally occurring and may act as seeds.^56^ From *in vivo* studies of Lewy bodies, truncations at residues 119 and 122 are most prevalent.^57−59^ Primary sites of metal ion binding have been localized to residues Asp121, Asn122, and Glu123 through heteronuclear single quantum coherence (HSQC) NMR experiments.^24^ It is thought that this region in the C-terminal domain might offer a favorable orientation of moieties for metal ion coordination governed by electrostatic interactions. The C-terminal region of full length αS has been suggested to exert a protective effect against aggregation due to the large number of negative charges which could form long-range interactions with the N-terminal region that shield the hydrophobic NAC region from nucleation events.^26^ Truncation of the C-terminal sequence has been shown in several studies to increase the rate of αS amyloid assembly, which we were also able to observe (Figure 6).^60−62^ Here, we explored whether divalent metal ions could exert a similar effect on the truncated, acetylated variant 119 of acceleration of amyloid formation despite some key divalent metal ion binding sites being deleted.

**Figure 7:**
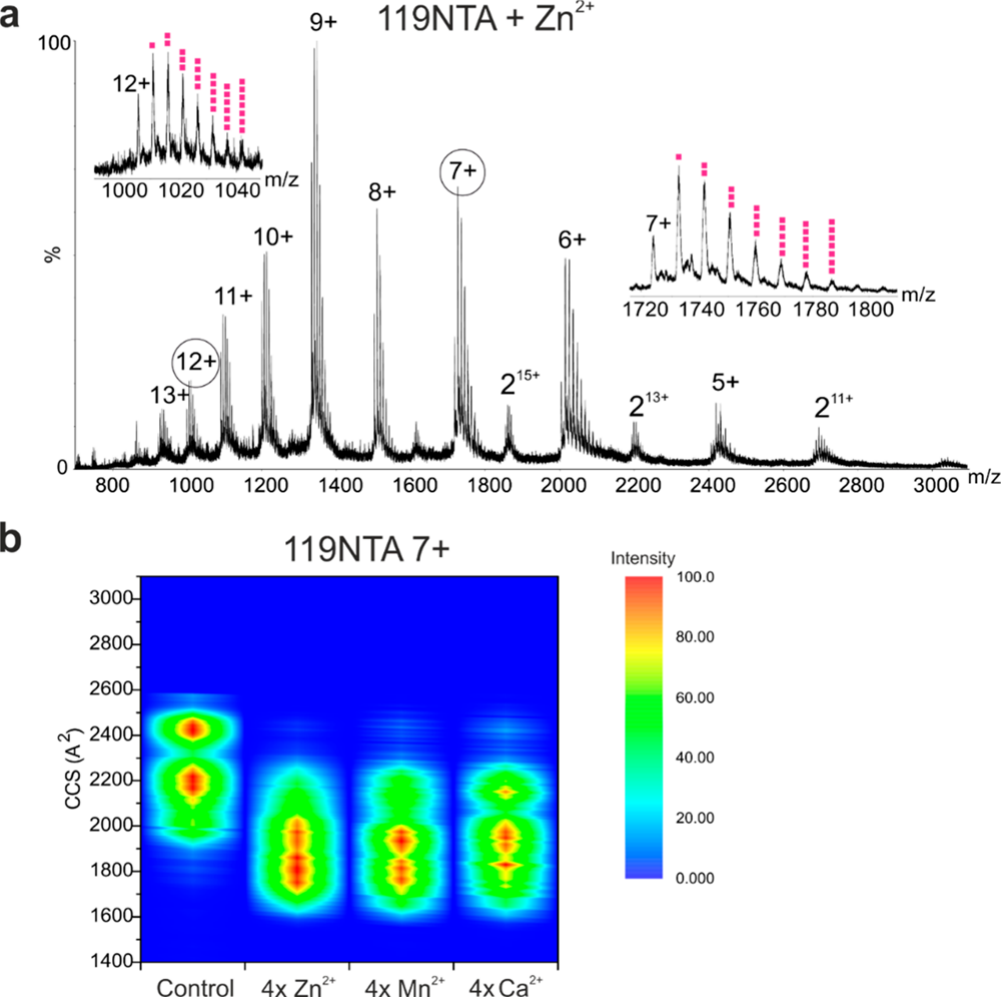
Native nESI-IM-MS spectra show compaction of 119NTA αS when metal ions bind. (a) Native nESI mass spectrum of 119NTA αS bound to Zn^2+^. Insets show up to seven Zn^2+^ ions bound to the 7+ and 12+ charge states (pink squares) (b) CCS fingerprints of 119NTA αS 7+ charge state alone or bound to four ions of either Zn^2+^, Mn^2+^, or Ca^2+^. All spectra were acquired using a protein concentration of 20 μM in 20 mM ammonium acetate, pH 7.5. Spectra with metal ions present were acquired in the presence of 500 mM ion acetate conjugate. All spectra were acquired on a Synapt G1 instrument. CCS values were calculated using ATDs extracted from MassLynx 4.1.

The effect of adding Zn^2+^, Mn^2+^, or Ca^2+^ to 119NTA αS on amyloid formation is shown in Figure 6. While Ca^2+^ results in comparable kinetics to the rate of amyloid assembly in the absence of metal ions, both Mn^2+^ and Zn^2+^ induce faster kinetics, despite the fact that the truncated variant forms amyloid already 5-fold more rapidly than WTNTA αS (Figure S6) and lacks two aspartic acid and six glutamic acid residues of the WT sequence. Native IM-MS of 119NTA αS [which displayed a multimodal charge state distribution in the absence of a metal ion (Figure S7)] shows that although eight D/E residues have been removed from the sequence, all three types of metal ions still bind to the protein sequence (Figure 7a and Figure S8), and all three ions shift the ensemble to populate compact conformations with the greatest conformational shift again observed in the presence of Zn^2+^ (Figure 7 and Figure S9). The N-terminal region, however, is unaffected by this deletion, with seven aspartic acid residues and one glutamic acid residue. These negative charges also offer essential binding sites for metal ions, which may have been overlooked in previous studies using very low concentrations of ions.^24^ Additionally to these residues, the N-terminal region contains two methionine residues which can act as interaction sites for transition metals.^25^ Furthermore, His50, which is removed in the ΔΔNTA variant of αS, may act as a compensatory metal ion binding site for 119NTA αS.

## Discussion

αS is an IDP, and its conformational behavior is known to be affected by divalent metal ion binding.^24,25,50,63,64^ Native IM-MS has shown that WTNTA αS populates four major conformational families at the 8+ charge state, consistent with previous data.^43,50^ We use CCS measurements to demonstrate that there is a clear and consistent link between the compaction of monomeric αS at the 8+ charge state for WTNTA αS and the 7+ charge state for ΔΔNTA αS with an increased rate of amyloid formation, possibly indicating that key species in the ensemble are compacted such that they are more competent to form amyloid. How the properties of species observed in the gas phase relate to those in solution (e.g., their hydrodynamic radius, or the formation of intra- or intermolecular contacts) will require further analyses using solution-based assays. For example, it is well-known that hydrophobic contacts are diminished in the absence of water, while hydrogen bonding and electrostatic interactions are enhanced.^65^ Such effects are especially important to consider for weak complexes, such as the early oligomers in amyloid formation, and dynamically disordered monomeric proteins, such as IDPs. Importantly, however, previous analyses comparing gas phase and solution properties of IDPs have shown that the ESI process does not have a substantial effect on structure and that IM-MS CCS can report accurately on their solution phase properties.^66^ Backed up with solution phase assays, therefore, MS-based methods provide a unique power for understanding the structure, populations, and stabilities of proteins in complex mixtures, as exemplified here for the effects of different metal ions in the self-assembly of αS. CCS values obtained also enable us to compare different deletion variants of αS in the absence of metal ions to determine the effect of the protein sequence and distribution of charged residues on the conformational behavior of the apo state. We show that WTNTA αS visits more conformational states than ΔΔNTA αS overall as shown in Figure 5, some of which presumably are competent to form amyloid. Shifting the conformational ensemble to populate species with smaller CCS combined with charge neutralization from divalent metal ion binding appear to have a key regulatory effect in increasing the rate of ThT positive amyloid assembly. Carija et al. used a disulfide link strategically placed between residues Val71 and Thr92 to lock the monomeric structure of WT αS into the Greek-key motif of αS amyloid fibrils. This compacted monomer resulted in reduced amyloid formation, highlighting the importance of conformational flexibility and dynamic conformational exchange in the early stages of assembly to generate the amyloid fold.^67^

There is a hierarchal reduction in the *t*_50_ of amyloid formation for WTNTA αS, whereby Zn^2+^ is the most effective, followed by Mn^2+^ and Ca^2+^. A study using laser ablation-inductively coupled plasma-MS (LA-ICP-MS) of homogenized human olfactory bulb samples from PD patients identified that Zn^2+^ was present at 10–200 μg/g concentrations, colocalized with aggregates of phosphorylated αS, while Mn^2+^ was found at trace levels (less than 1 μg/g on average). Zinc in particular was thought to contribute to Lewy body pathology in PD through oxidative stress, having a mostly pathological role.^47^ Physiological Ca^2+^ concentrations can vary from tens of nM to several hundreds of μM, depending on whether neuronal cells are in a resting state or undergoing depolarization during an action potential.^68^ With a *K*_D_ of 21 μM for αS with Ca^2+^, it is clear that αS will interact with Ca^2+^ in the physiological, cellular context.^42^ Lautenschläger et al. showed a relationship between C-terminal Ca^2+^ binding and αS synaptic vesicle interactions.^42^ Moreover, the presence of Ca^2+^ ions increased the affinity of αS to synaptic vesicle membranes, which resulted in increased clustering of vesicles.^42^ The presence of metal ions influences the behavior of αS within cells and may elude to mechanisms hidden in synucleopathies. This study provides further molecular detail into the resulting effects on αS when metal ions bind.

*In vivo*, binding of other small molecules or proteins such as chaperones may offer a protective effect against pathological structural remodelling of αS. Molecules such as dopamine are known to also bind to the C-terminal region of αS, and binding results in extension of the protein chain which offers a protective effect, switching off aggregation into the amyloid.^69^ Additionally, chaperones such as Hsp70 and Hsp40 are known to interact with the N-terminal region of αS, protecting it from amyloid assembly.^70,71^*In vivo*, chaperone binding could offer a protective effect which counteracts the effects of metal ion binding.

Introducing the ΔΔ variant to the αS sequence through removal of P1 and P2 sequences switches off the potential for αS to assemble into amyloid by altering the long-range interactions necessary to induce an amyloid-competent conformation.^34^ Even though these sequences remove critical interaction sites, we show that ΔΔNTA αS still remains conformationally dynamic by native IM-MS (Figure 4b and Figure S2b). However, this variant populates three distinct conformations at the 7+ charge state wherein the largest CCS conformation (most extended) is the most intensely populated, whereas WTNTA αS populates four CCS conformations (at the 8+ charge state) with the population intensity weighted toward the compact conformations. Strikingly, when divalent metal ions are present, the ΔΔNTA αS amyloid assembly potential is re-established, and this effect consistently correlates with compaction, reinforcing the correlation between chain compaction and the rate of amyloid formation.

As metal ion binding is believed to be located primarily to negatively charged Asp and Glu residues in the C-terminal region of αS, physiologically relevant C-terminally truncated variants of αS may inform on the necessity of charge neutralization in this process, or whether compaction alone is the primary driver. Additionally, transition metals can also interact with methionine and histidine residues, potentially recruiting the N-terminal region of αS; however, this is not known for Ca^2+^.^24,25,42^ We repeated native IM-MS and ThT kinetics on a variant of αS truncated at residue 119 (1–119), which was also acetylated at the N-terminus (119NTA) in the absence and presence of metal ions. Native IM-MS in Figure 7 shows that the +7 charge state of 119NTA αS variant populates fewer conformational states than ΔΔNTA αS, where the intensity is split between one extended conformation and one compact conformation with some additional compact CCS values represented as weaker intensity below the intense compact conformation CCS. Interestingly, despite half of the putative C-terminal metal binding region being removed in 119NTA αS, an almost comparable number of ions still bind to the protein, with 1–2 fewer binding events on average compared to WTNTA αS. These binding events still result in CCS compaction and faster amyloid assembly kinetics. These results imply that the N-terminal region must also be important for metal ion binding and that the presence of a compact conformation is compatible with metal ion binding. The seven aspartic acid residues and single glutamic acid residue within the N-terminal region may become preferential to divalent metal ions when the C-terminal region is truncated. His50 may offer an essential binding mechanism which anchors metal ions by coordination with the C-terminal region creating a seemingly looped structure of αS.

Compact conformations may offer a preferential, higher stability orientation for metal ion binding as the close proximity of negatively charged amino acids creates a binding pocket when the C-terminus folds backward to interact with the N-terminus. Ions can create a coordination network between these negatively charged amino acids which pull the structure into a tighter, more compact conformation which may also result in increasingly rigid conformations, depicted from low charge states in the CCS evaluation in Figure 5. CCS compaction has been observed previously in the case of increased numbers of Ca^2+^ binding to calmodulin, established by IM-MS.^72^ As for the higher charge states, these extended conformations still bind to Zn^2+^ and other metal ions; however, due to the distance between negatively charged residues, metal ions presumably cannot hold distant residues together, resulting in no significant observable conformational change.

## Conclusion

We hypothesize that the conformational ensemble of the monomeric state predisposes α-synuclein’s functional and amyloid-forming behavior. The charge distribution across the sequence of the protein might be naturally fine-tuned in a way that prevents the rapid onset of amyloid assembly, tipping the toxicity versus function of αS away from toxicity. Binding of metal ions or other ligands such as small molecules, lipids, or membranes could bias αS toward amyloid-prone conformations, resulting in the rapid assembly of toxic oligomers and ultimately amyloid fibrils which are associated with disease.

Further information regarding the exact binding sites of metal ions to WTNTA αS, as well as ΔΔNTA αS and 119NTA αS, will help to elucidate the exact molecular rearrangement that the αS protein chain undergoes when metal ions bind. Binding sites can be mapped using techniques such as native top-down electron-capture or transfer dissociation (ECD/ETD) tandem mass spectrometry (MS/MS) which fragments the backbone of intact proteins while maintaining noncovalent ligand binding. In addition, residue- and region-specific details of the conformational changes that αS undergoes in the presence of metal ion binding could be unravelled using techniques such as NMR or cross-linking MS to identify specific intraprotein interactions that stabilize compact conformations or studies using techniques such as smFRET analysis of population shifts using probes placed at relevant sites on the protein sequence.^73^ With powerful MS methods adding to a fast-growing structural toolbox, our next goal is to target the structural gap in protein aggregation pathways between monomer and fibril, by elucidating structural intermediates such as oligomeric and phase-separated states and identify potential targets for pharmaceutical intervention.

## Supporting Information Available

*t*_50_ values and fibril yields for WTNTA and ΔΔNTA αS amyloid assembly as well as 119NTA αS amyloid assembly, native ESI mass spectra showing unbound WTNTA and ΔΔNTA αS as well as Mn^2+^ and Ca^2+^ binding to WTNTA αS and ΔΔNTA αS, native IM mass spectra showing compaction of WTNTA, ΔΔNTA αS, and 119NTA αS when different numbers of Zn^2+^ ions bind, and native ESI mass spectra showing 119NTA αS in the absence of metal ions as well as Mn^2+^ and Ca^2+^ binding to 119NTA αS (PDF)
