# Supplementary material for: Taking Charge: Metal Ions Accelerate Amyloid Aggregation in Sequence Variants of α-Synuclein
Source: J Am Soc Mass Spectrom. 2023 Feb 16;34(3):493–504. doi: 10.1021/jasms.2c00379 (PMC9983014; doi:10.1021/jasms.2c00379)
Supplement: Supplementary file 1 — js2c00379_si_001.pdf [file js2c00379_si_001.pdf]

Supporting Information

Taking Charge: Metal ions accelerate amyloid aggregation in sequence variants of α-synuclein

Emily J. Byrd[1], Martin Wilkinson[1], Sheena E. Radford[1], Frank Sobott[1*]

[1]Astbury Centre for Structural Molecular Biology, School of Molecular and Cellular Biology, Faculty of Biological Sciences, University of Leeds, Leeds, LS2 9JT, United Kingdom.

[*]Correspondence: f.sobott@leeds.ac.uk

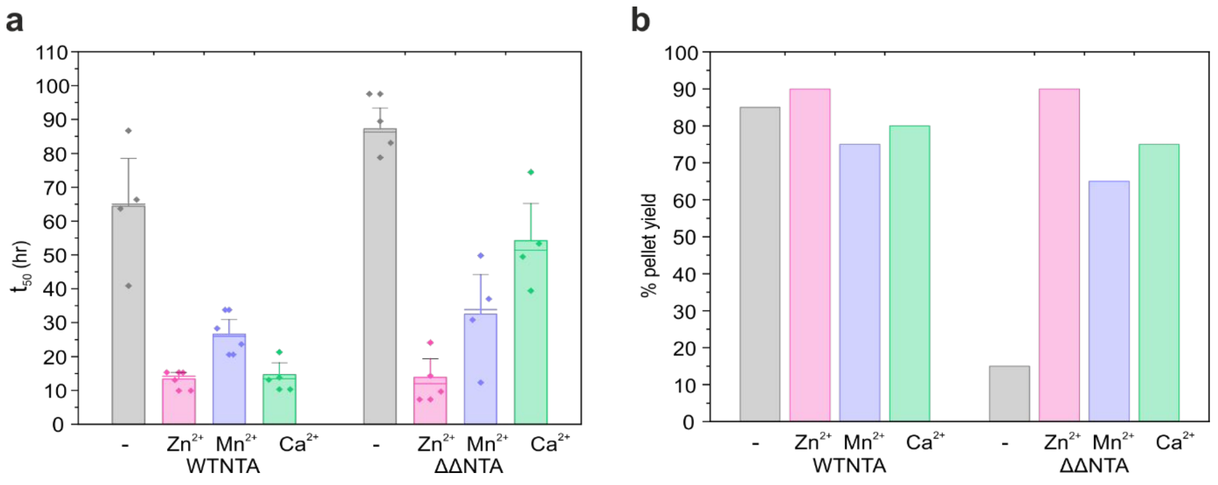

Figure S1: t_{50} values and fibril yields for WTNTA and ΔΔNTA αS amyloid assembly. *(a) t_{50} values for the rate of amyloid assembly for WTNTA αS and ΔΔNTA αS, determined from ThT plate assays. Each condition was measured in at least triplicate. Error bars show the standard deviation of the mean of the replicates. (b) Fibril yields of pelletable material from the end-points of amyloid assembly for WTNTA αS and ΔΔNTA αS. Given errors in estimating pellet yields via SDS PAGE subsequent to centrifugation (see Methods), the values were rounded to the nearest 5%. Single pelleting experiments were performed here and hence errors are not shown.*

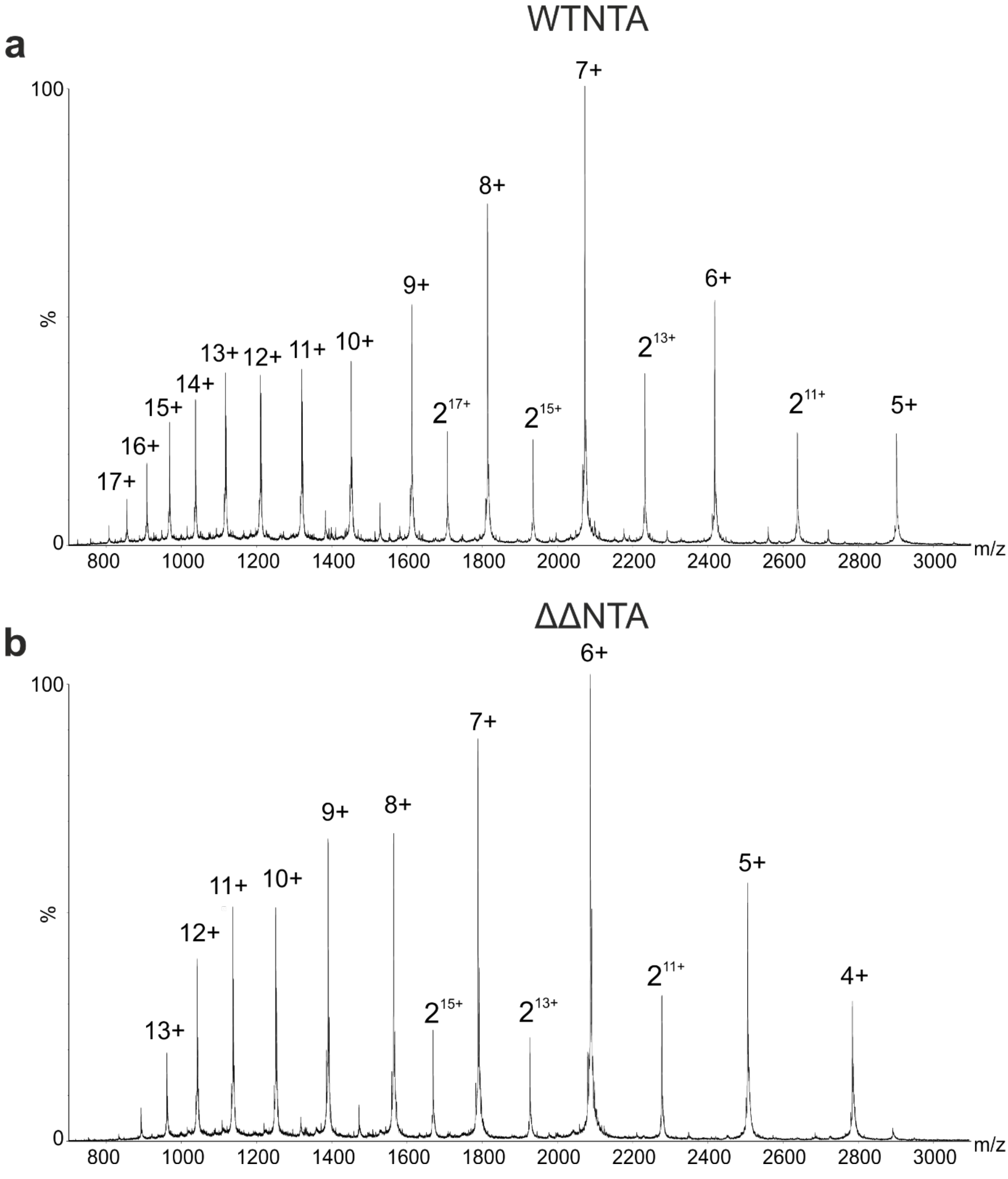

Figure S2: Native ESI mass spectra showing unbound WTNTA and ΔΔNTA αS. *(a) The native nESI mass spectrum of WTNTA αS. (b) As in (a), but for ΔΔNTA. The protein concentration was 20 µM in 20 mM ammonium acetate pH 7.5. Dimers are indicated by '2'.*

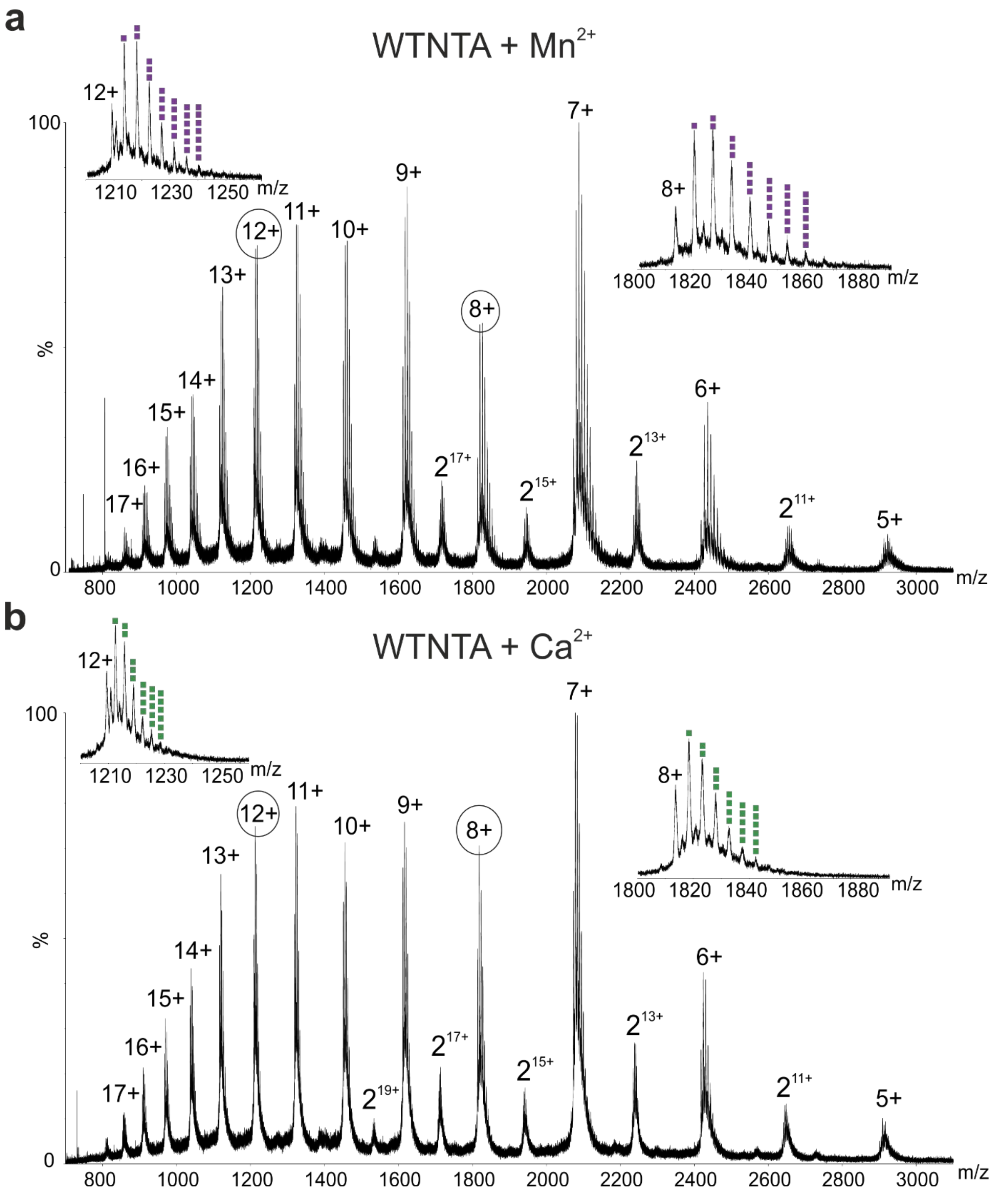

Figure S3: Native ESI mass spectra showing Mn^{2+} and Ca $^{2+}$ binding to WTNTA αS. *(a) The native nESI mass spectrum of WTNTA αS bound to Mn^{2+} ions. The insets show up to seven Mn^{2+} ions bound to the 8+ and 12+ charge states (purple squares). (b) As in (a), but for Ca^{2+} binding. The insets show up to six ions bound to the 8+ and 12+ charge states. The protein concentration was 20 µM in 20 mM ammonium acetate pH 7.5 and a molar ratio of 1:25 αS:metal ion was used. Dimers are indicated by '2'.*

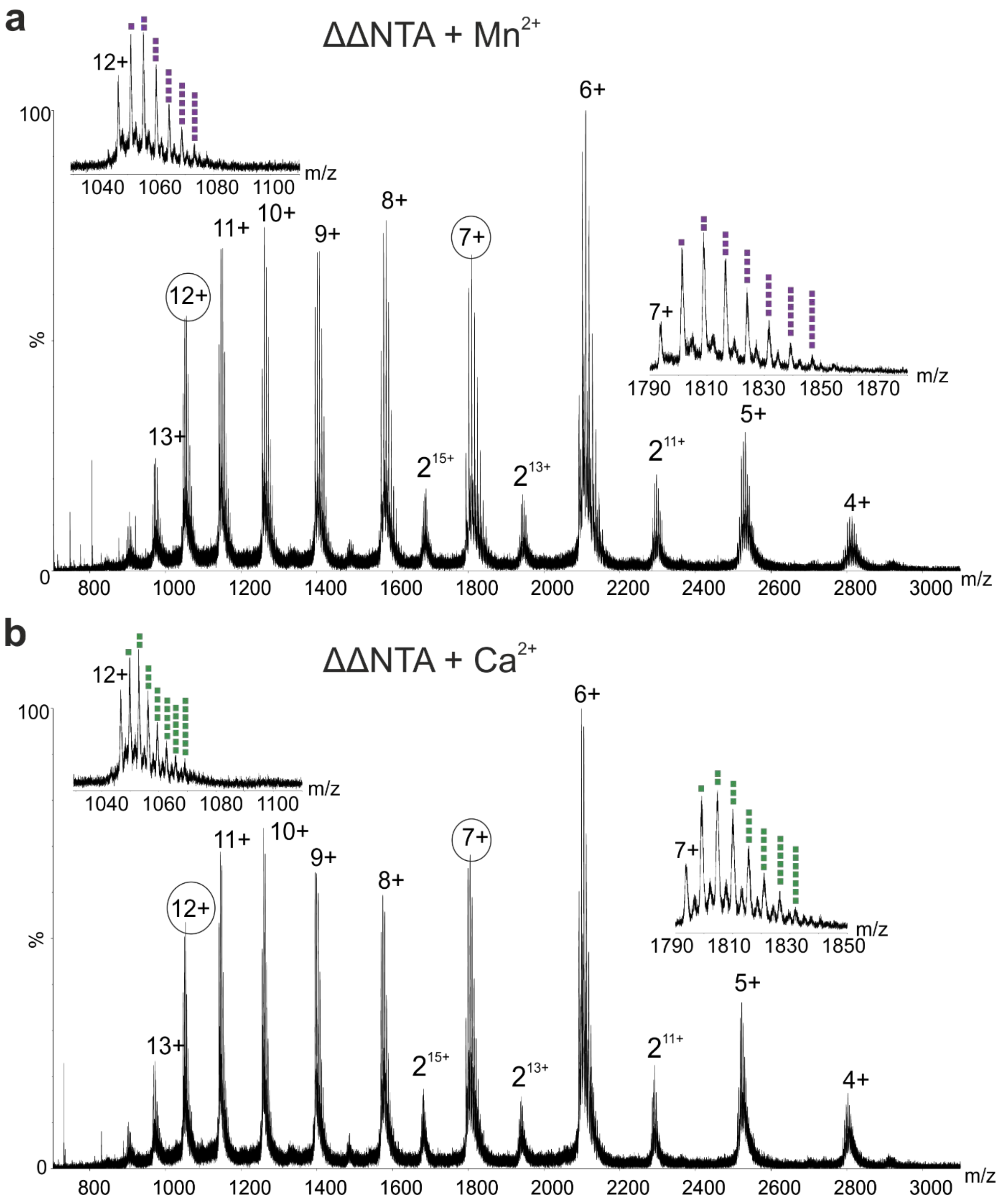

Figure S4: Native ESI mass spectra showing Mn^{2+} and Ca^{2+} binding to ΔΔNTA αS. *(a) The native nESI mass spectrum of ΔΔNTA αS bound to Mn^{2+} ions. The insets show up to six-seven Mn^{2+} ions bound to the 7+ and 12+ charge states (purple squares). (b) As in (a), but for Ca^{2+} binding. The insets show up to seven ions bound to the 7+ and 12+ charge states. The protein concentration was 20 µM in 20 mM ammonium acetate pH 7.5 and a molar ratio of 1:25 αS:metal ion was used. Dimers are indicated by '2'.*

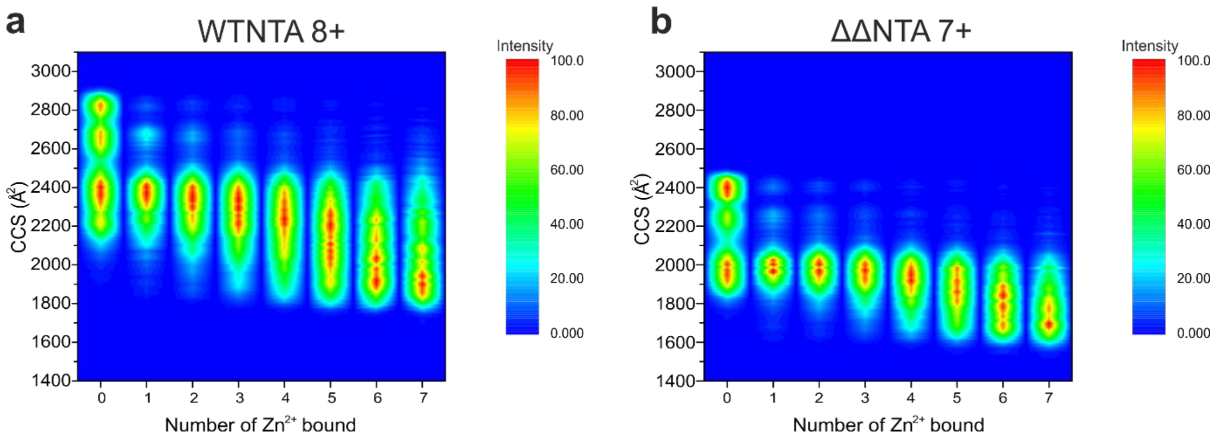

Figure S5: Native IM mass spectra showing compaction of WTNTA and ΔΔNTA αS when Zn^{2+} ions bind. *(a) CCS fingerprints of the 8+ charge state of WTNTA αS unbound and bound to one to seven Zn^{2+}. (b) CCS fingerprints of the ΔΔNTA αS 7+ charge unbound and bound to one to seven Zn^{2+}. All spectra were acquired using a protein concentration of 20 µM in 20 mM ammonium acetate, pH 7.5. A 25-fold molar excess of zinc acetate was added. CCS values were calculated using ATDs extracted from MassLynx 4.1 software and calibrated as described in the Methods section.*

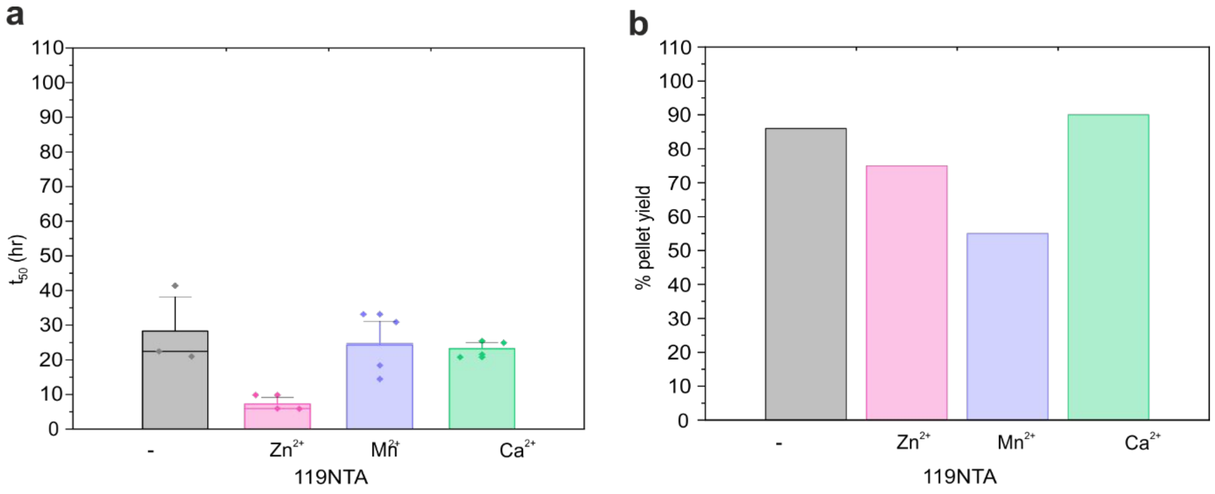

Figure S6: t_{50} values and fibril yields for 119NTA αS amyloid assembly. *(a) t_{50} values for the rate of amyloid assembly for 119NTA αS, determined from ThT plate assays. Each condition was measured in at least triplicate. Error bars show the standard deviation of the mean of the replicates. (b) Yields of pelletable material from the end-points of amyloid assembly for 119NTA αS. Values are rounded to the nearest 5%. Single pelleting experiments were performed here and hence errors are not shown.*

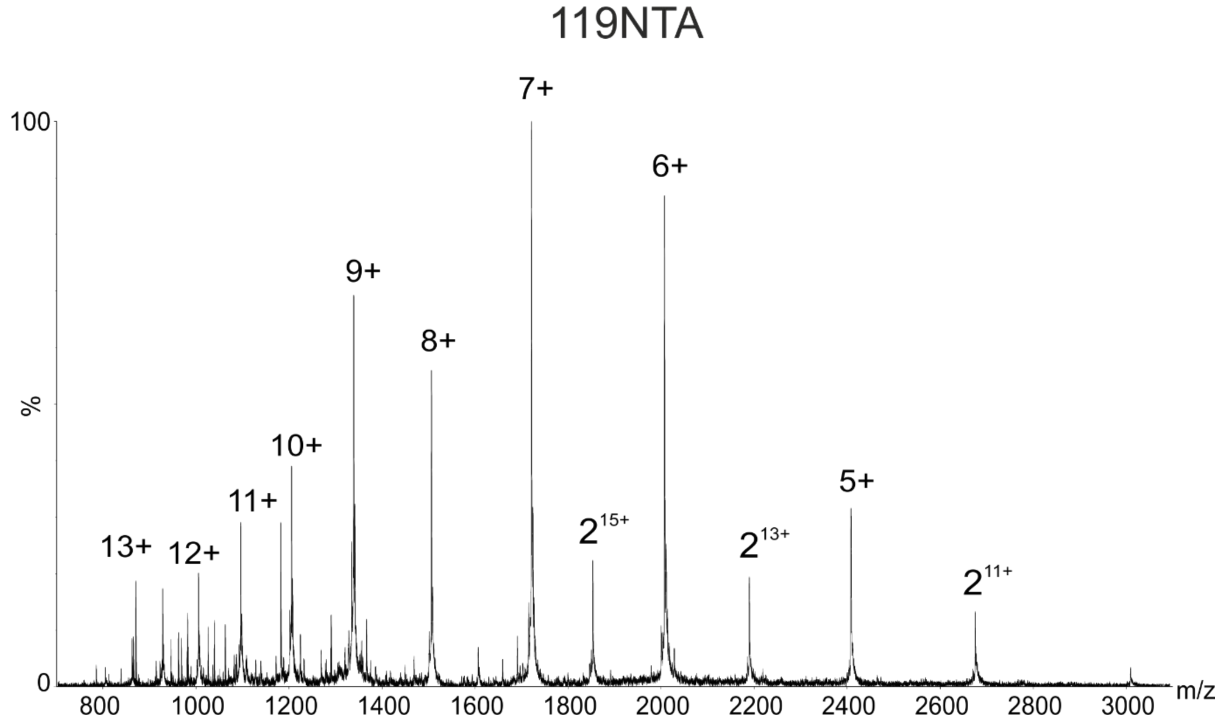

Figure S7: Native ESI mass spectra showing 119NTA αS in the absence of metal ions. *The native nESI mass spectrum of 119NTA αS. The protein concentration was 20 µM in 20 mM ammonium acetate pH 7.5. Dimers are indicated by '2'.*

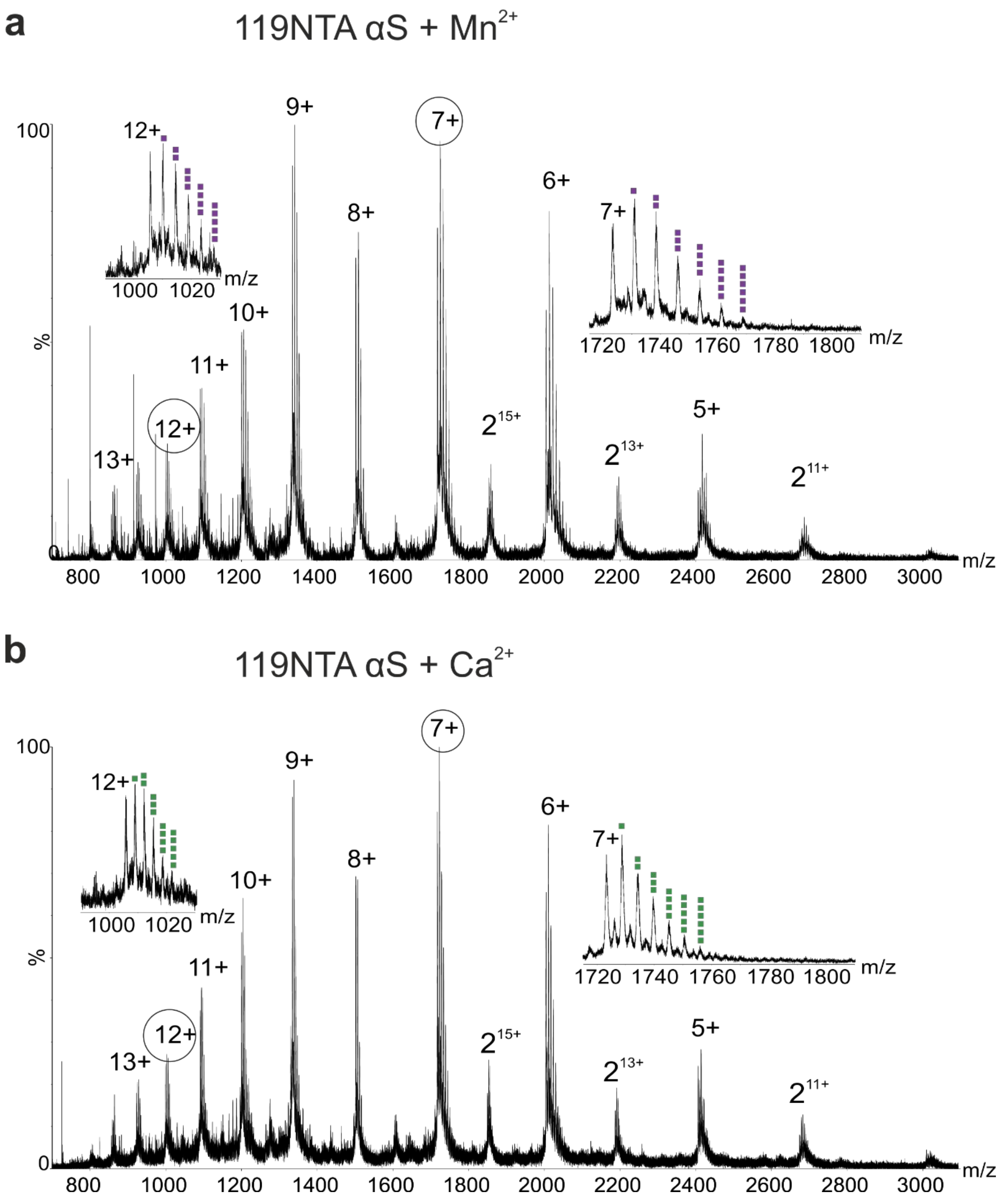

Figure S8: Native ESI mass spectra showing Mn^{2+} and Ca $^{2+}$ binding to 119NTA αS. *(a) The native nESI mass spectrum of 119NTA αS bound to Mn^{2+} ions. The insets show up to five and six Mn^{2+} ions bound to the 12+ and 7+ charge states respectively (purple squares). (b) As in (a), but for Ca^{2+} binding. The insets show up to five and six ions bound to the 12+ and 7+ charge states respectively. The protein concentration was 20 μM in 20 mM ammonium acetate pH 7.5 and a molar ratio of 1:25 αS:metal ion was used. Dimers are indicated by '2'.*

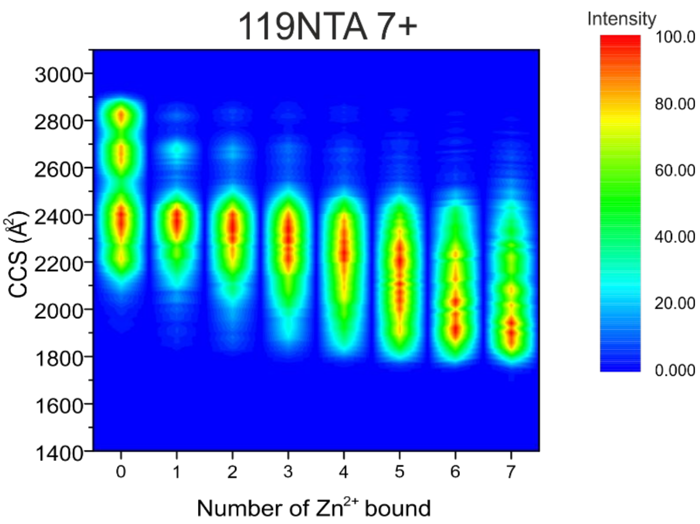

Figure S9: Native IM mass spectra showing compaction of 119NTA αS when Zn^{2+} ions bind. *CCS fingerprints of the 7+ charge state of 119NTA αS unbound and bound to one to seven Zn^{2+}. All spectra were acquired using a protein concentration of 20 µM in 20 mM ammonium acetate, pH 7.5. A 25-fold molar excess of zinc acetate was added. CCS values were calculated using ATDs extracted from MassLynx 4.1 software and calibrated as described in the Methods section.*
